# A systematic review of cardiac time intervals utilising non-invasive fetal electrocardiogram in normal fetuses

**DOI:** 10.1186/s12884-018-2006-8

**Published:** 2018-09-12

**Authors:** Vinayak Smith, Senthuran Arunthavanathan, Amrish Nair, Diane Ansermet, Fabricio da Silva Costa, Euan Morrison Wallace

**Affiliations:** 1 Department of Obstetrics and Gynaecology, Monash University, 252 Clayton Road, Melbourne, VIC 3168 Australia; 2 Department of Electrical and Electronic Engineering, University of Melbourne, Parkville Campus, Melbourne, VIC 3010 Australia; 3 Biorithm Pte Ltd, 81 Ayer Rajah Crescent 03-53, Singapore, 139967 Singapore

**Keywords:** Fetal electrocardiogram, Cardiac time intervals, Non-invasive fetal monitoring

## Abstract

**Background:**

Non-invasive fetal electrocardiogram (NIFECG) is an evolving technology in fetal surveillance which is attracting increasing research interest. There is however, only limited data outlining the reference ranges for normal cardiac time intervals (CTIs). The objective of our group was to carry out a systematic review to outline normal fetal CTIs using NIFECG.

**Methods:**

A systematic review of peer reviewed literature was performed, searching PUBMED,Ovid MEDLINE and EMBASE. The outcomes of interest included fetal CTIs (P wave duration, PR interval, QRS duration and QT interval) and a descriptive summary of relevant studies as well. The outcomes were grouped as early pre-term (*≤* 32 weeks), moderate to late pre-term (32–37 weeks) and term (37–41 weeks).

**Results:**

8 studies were identified as suitable for inclusion. Reference ranges of CTIs were generated. Both PR interval and QRS duration demonstrated a linear correlation with advancing gestation. Several studies also demonstrated a reduction in signal acquisition between 27 and 32 weeks due to the attenuation by vernix caseosa. In this group, both the P wave and T waves were difficult to detect due to signal strength and interference.

**Conclusion:**

NIFECG demonstrates utility to quantify CTIs in the fetus, particularly at advanced gestations. Larger prospective studies should be directed towards establishing reliable CTIs across various gestations.

## Background

Evaluation of the fetal cardiac activity remains a cornerstone of obstetric practice. These are broadly classified into invasive and non-invasive forms of monitoring. At present, the main modalities being utilised include:

### Cardiotocography

Cardiotocography (CTG) is a non-invasive form of monitoring which has been utilised widely to measure the fetal heart rate (FHR) by means of Doppler ultrasound since the 1970s. Through this, an ultrasound wave of 1.5 MHz is utilised to resonate with the fetal cardiac structures. Subsequently, the dispersed waveforms are measured through a transducer via the ensuing Doppler effect. This produces an approximation of the fetal heart rate using autocorrelation techniques which compare and average it against the previous doppler waveforms. The methodology however, is not without its shortcomings [1]. Several issues with respect to the data acquired have been outlined which include the lack of beat to beat data (i.e. true fetal heart rate variability), signal loss during monitoring (15–40%), signal artifacts (i.e. resulting in double and half counting), the inability to detect fetal arrythmias and confusion between maternal heart rate (MHR) and FHR [2–4]. These limitations are generally well appreciated by the workforce, obstetricians and midwives, but significant mis-interpretation and harm continues to arise from them. From the perspective of the pregnant woman herself, CTG technology appears cumbersome, limiting mobility even where wireless transducers are employed [5].

### Direct fetal electrocardiogram (FECG)

One approach to improving signal detection is the use of the fetal scalp electrode (FSE). This involves the direct application of an electrode to the fetal scalp and requires adequate cervical dilation as well as rupture of the amniotic membranes. This method provides a more reliable measurement of the FHR than indirect CTG. It generates a FHR by identifying the R-R interval (separation between an R peak and the following R peak) on the FECG signal. Given its invasive nature, it can only be used in labour and cannot be applied antenatally. Further, there is a small risk of injury to the fetal scalp and use of the FSE is relatively contraindicated in preterm infants and contraindicated in fetuses with bleedings disorders, instances of maternal viremia (such as Hepatitis B/C/D/ E as well as Human Immunodeficiency Virus) and chorioamnionitis [2, 6].

Both CTG and FSE are predominantly focused on screening for FHR changes secondary to fetal hypoxia to guide obstetric decision regarding timing of delivery and thereby preventing fetal injury, particularly brain injury. Unfortunately, the results from randomised controlled clinical trials do not demonstrate that the use of CTGs, at least in low risk births, has improved perinatal mortality or longer term outcomes associated with injury, such as cerebral palsy. On the contrary, increasing uptake of CTGs has been associated with an increase in obstetric intervention [7].

### Non-invasive fetal electrocardiogram (NIFECG)

An alternative form of monitoring which has been recently gaining increasing attention involves utilising NIFECG. This is carried out by using surface electrodes on the abdomen of the pregnant woman and obtaining a FECG signal.

The NIFECG promises to offer assessment of both the FHR rhythm as well as its morphology. Additional information such as fetal orientation and movements assessment can be garnered from the signal too [5]. With these advances, NIFECG potentially offers a superior quality of FHR information in comparison to existing monitoring modalities such as CTG or FSE [8, 9]. NIFECG has also the ability to reduce MHR and FHR confusion and to monitor women with a high body mass index (BMI) more effectively [10–12].

Cardiac time interval (CTI) analysis is a core in the evolving field of morphology analysis that is attracting an increasing amount of research. CTIs specifically refer to the duration of the P wave, PR interval, QRS complex and QT interval. Amongst its utilisation in detecting hypoxia, novel indications linked to its utility include screening for congenital heart defects, determining true heart rate and short term variability, fetal arrhythmias and fetal growth restriction as well [6, 13, 14].

As with other forms of monitoring however, there have been some technological challenges related to detection of the NIFECG. Primarily, this has been related to the low signal-to-noise ratio (SNR) of the FECG signal, due to its low electrical amplitude (1*/*50 that of the maternal ECG signal) and the large maternal ECG (MECG) and background noise [5, 15]. In addition, there are some limitations to signal acquisition as well. For instance, between the 27th to 32nd week of gestation, signal acquisition becomes more onerous due to the attenuation caused by the vernix caseosa surrounding the fetus [16] . Another important limitation is the lack of available references and databases for comparison of NIFECG signal [5, 6]. Nevertheless, recent signal processing techniques and advances in data processors have afforded improved consistency in signal acquisition and analysis [5].

### Objective

Considering these observations, the following review aims to provide a reference for researchers in the field to identify the normal range of CTIs for fetuses across a range of gestational ages when utilising NIFECG. CTIs vary through gestation and available information is limited by the finite number of observational studies addressing this topic. In addition, correlation between the CTIs and end-points in both animal models and humans are presented in the discussion to provide a guide on end-points which ongoing research can be directed towards as well.

## Data sources

**Fig. 1:**
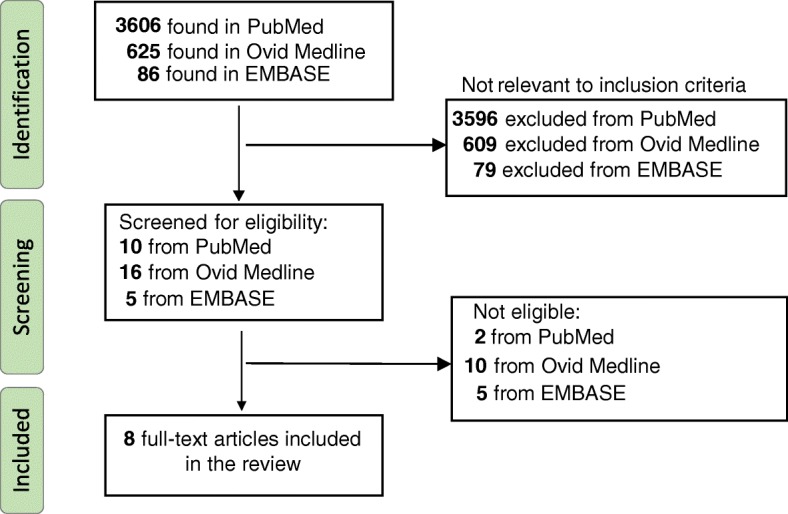
Search strategy for the systemic review

The inclusion criteria for the following study are studies examining the CTIs in fetuses with normal cardiac anatomy with the utilisation of NIFECG technology specifically across all gestational ages. CTIs of interest would include the P wave, PR interval, QRS complex and QT interval. The ST segment will not be included in this review as it is not a routine part of CTI analysis, has only preliminary data proving its feasibility and is more in relation to morphological analysis of the waveform [17–19]. No limitation was placed on the year of publications or on language. For the following systematic review,

PubMed, Ovid MEDLINE and EMBASE were searched to identify studies till 30th of May 2018. by DA and VS. The following keywords were utilised for the search, utilising both British and American spellings: “fetal electrocardiogram”, “fetal ECG”, “fetal electrocardiography” and “fetal cardiac time intervals”. The database was searched independently by VS and DA. The reference lists of articles which were identified were also searched. Review articles as well as articles focusing on other techniques other than NIFECG were excluded. Once articles were identified, the ones suitable for the review were selected by consensus between DA, AN, and VS. The search strategy for the study is presented in Fig. 1.

### Data collection process

Data on fetal CTIs were extracted from the individual studies manually for analysis. Due to the varied methods of signal acquisition, processing and CTI estimation used between studies, data could not be pooled together for meta-analysis. In addition, descriptive analysis of the studies was carried out to describe their characteristics and provide quality assessment.

### Data items

Data of interest included assessment of the quality of the study and factors affecting signal acquisition as well as processing. For the fetal CTIs in particular (P wave duration, PR interval, QRS duration and QT interval), importance was given to examining their duration across various gestations and appreciating the success rates in acquiring them as well. Particular attention was paid to assessing the presence of signal attenuation between 27 to 32 weeks as quoted in the literature.

### Assessment of Bias

**Fig. 2:**
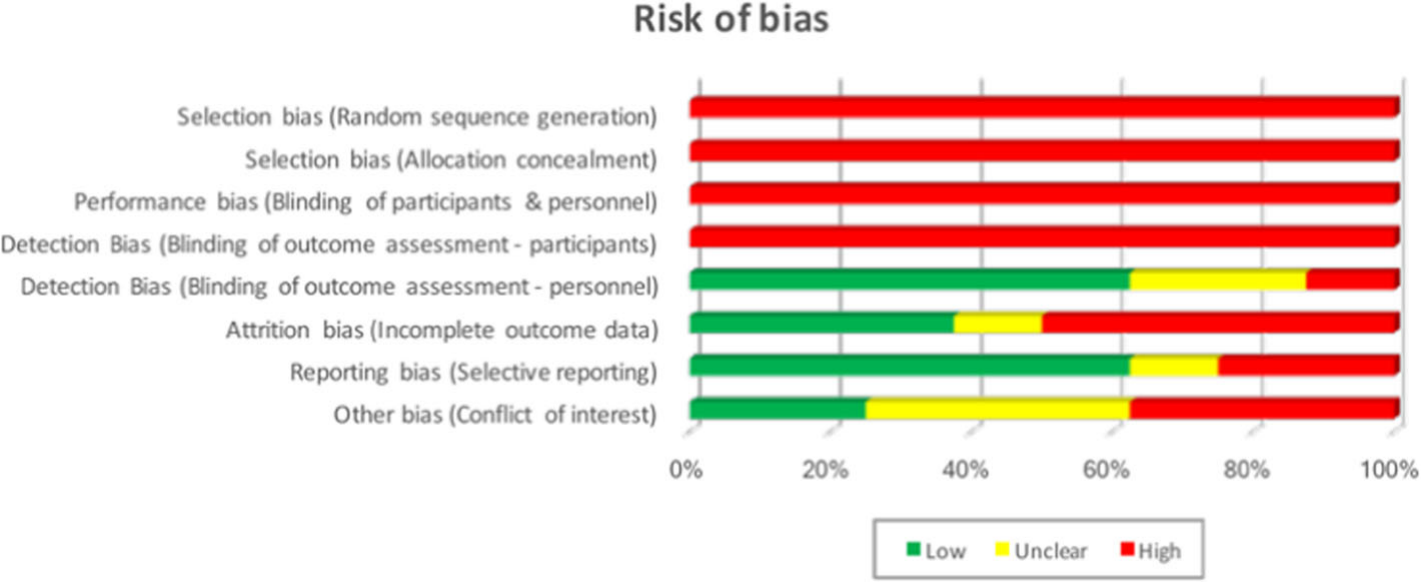
Graphical representation of the risk of bias as per the Cochrane Tool. Results are presented as a percentage across all studies (*n* = 8)

The risk of publication bias was assessed by DA and VS utilising the Cochrane tool and is illustrated in Fig. 2.

### Summary measures and synthesis of results

The compiled information regarding fetal characteristics are presented in a tabular form in terms of descriptive summary, CTI data, and signal acquisition.

#### Descriptive summary of results

Each paper was reviewed manually by VS, DA and AN and information was compiled in consensus.

#### CTI data

Each paper was reviewed manually by VS and SA and information was compiled in consensus.

The data acquired from the studies were noted to be of varying gestational ages (GA). To provide a clinically relevant overview of all the data, the findings were grouped in the manner of early pre-term (≤ 32 weeks), moderate to late pre-term (32–37 weeks) and term (37–41 weeks) as per the World Health Organization (WHO) Classification.

An overview of how data was handled for each study is presented below:

- “Abboud 1990”: The CTI parameters are presented between the GA of 36 to 41 weeks. The average PR intervals, QRS durations, and QT intervals are calculated based on this and classified into late pre-term and term [20] .
- “Arya 2015”: The CTI parameters are presented for the GAs ranging from 19 to 39 weeks as individual cases. We have only included foetuses with normal cardiac anatomy in the following analysis. The cases have been reclassified into early pre-term and late pre-term from which the averages of the PR intervals, QRS durations, and QT intervals are calculated [21].
- “Chia 2005”: The CTI parameters are presented for the GA of 18–22, 23–27, 28–32, 33− ≤ 37, ≥ 37 weeks in the study. These GA periods have been reclassified into early pre-term, late pre-term, and term from which the values have been averaged [22].
- “Hayashi 2009”: The QRS durations for the GA periods ranging from 32 to 35 weeks and ≥ 36 weeks. These have been reclassified as late pre-term and term which allows averaging of the QRS values [23] .
- “Taylor 2003”: The predicted PR intervals, QRS durations, and QT intervals for the GAs of 20, 30, and 40 are presented. Such GA intervals are provided

as the information during the intervals of 20–30 and 30–40 are not provided in a tabular format but graphical form. The GA of 30 and 40 are selected and recategorized into early pre-term and late pre-term and term [24].

- “Taylor 2005”: The PR intervals, QRS durations, and QT intervals for GAs ranging from 24 to 41 weeks are selected as cases. These studies are re classified into early pre-term and term for the CTIs [25].
- “Wacker-Gussmann 2017”: The FECG findings for the GA periods of 32–33, 34–35, 36–37, and 38–40 are presented. The findings are averaged and reclassified into late pre-term and term [26].
- “Yilmaz 2015”: The PR intervals and QRS durations for the GA of 20–24, 28–32, and 34–38 weeks are reclassified as early and late pre-term. We have only included foetuses with normal cardiac anatomy in the following analysis. [27].

#### Signal acquisition

Each paper was reviewed manually by VS, SA and information was compiled in consensus in terms of signal acquisition loss between 27 and 32 weeks of GA.

## Results

A summary of the characteristics of each study is presented in Table 1. Table 1 provides a descriptive analysis of the studies included in the systematic review. The characteristics have been differentiated into study related information, signal acquisition and signal processing methods. Of note are the differences in time intervals examined, signal acquisition and signal processing methods. These variances contribute to the results being independent and limiting the ability of the data to be pooled together.

**Table 1:** Descriptive summary of studies included in review

Characteristics	Abboud et al.	Taylor et al.	Chia et al.	Taylor et al.	Hayashi et al.	Arya et al.	Yilmaz et al.	Wacker-Gussman. et al.
Study design and methods								
Year of Publication	1990	2003	2005	2005	2009	2015	2015	2017
Study design	Cross sectional study	Cross sectional study	Prospective cohort study	Cross sectional study	Cross sectional study	Cross sectional study	Prospective observation cohort study	Cross sectional study
Inclusion criteria	None reported	Random sampling	Healthy women and fetuses only	Women in labour or threatened labour	None reported	Referred for fetal echocardiography	Fetuses with normal cardiac anatomy or CHD	Healthy fetuses only
Gestational Age [weeks]	32 to 41	15 to 41	18 to 41	24 to 41	32 to 41	16 to 42	20 to 38	32 to 40
Time interval examined	PR, QRS, QT	PR, QRS, QT, QTc	P, PR, QRS, QT, T	PR, PQ, QRS, QT, QTc	P, QRS, T	PR, QRS, RR, QT	PR, QRS, QT	PR, PQ, QRS, QT, QTc
Number of patients	Not described	241	100	15	48	50	92	149
Number of acquired FECG	21	250	374	15	48	60	177	149
Signal acquisition								
Number of electrodes	2	14	3	14	5	5	5	5
Electrodes placement	Not described	12 evenly over abdomen, 1 ground ref. near navel, 1 common ref. on right ankle	Equilateral triangle formation: 1 right + 1 left hypochondriac area, 1 above symphysis pubis	12 evenly over abdomen, 2 references near umblicus	2 higher abdomen (right/left), 2 lower abdomen (right/left), 1 at distinct fetal heart sound location	2 along midline, 2 at each side of uterus, 1 on left flank	2 along midline, 2 at each side of uterus, 1 on left flank	2 along midline, 2 at each side of uterus, 1 on left flank
Dermabrasion & impedance	None described	Yes (*<* 5 kΩ)	Yes(*<* 5 k Ω)	Yes (*<* 5 kΩ)	None reported	Yes	Yes	Yes
Reported difficulty in signal acquisition	None reported	Between 27 and 36 weeks	Between 27 and 32 weeks	None reported	None reported	Between 26 and 30 weeks	Between 24 and 34 weeks	Lowest at 32 weeks
Signal processing								
FECG analysed	21	199	374	12	35	20	132	117
Signal averaging for analysis	100 beats	1 min	2.5 min	1 min	300–500 beats	1 min	yes - not described	1000 beats
Digital filters	4-pole Butterworth non-recursive 10 Hz Highpass	1–2 Hz Highpass, 150 Hz lowpass	1st & 2nd derivative template removal	1 Hz Highpass, 150 Hz Low pass	0.5 Hz low-level an 25 Hz high level removal	[0*.*5–70 Hz] Butterworth bandpass	Automated with Monica DK V1.8 software	Not reported
MECG removal	Digital subtraction of averaged signal	See Patent appl. GB2002/004410	Template subtraction	See Patent appl. GB2002/004410	Template subtraction	Template matching and digital subtraction	Automated with Monica DK V1.8 software	Template subtraction
Success rates in CTI detection	QT-80%	P,Q, R, S-100%; T-78%	P-74.6%, QRS-91.0%, T-79.3%	P, QRS-100%, T-92%	72.9%	100% due to strict FECG excl. Criteria	PR-77%, QRS-98%, QT-30%	P, PQ, PR-97%, QRS-100%, T-41%

*CTI* Cardiac time intervals, *FECG* fetal electrocardiogram, *MECG* Maternal electrocardiogram

### Normal CTIs

**Fig. 3:**
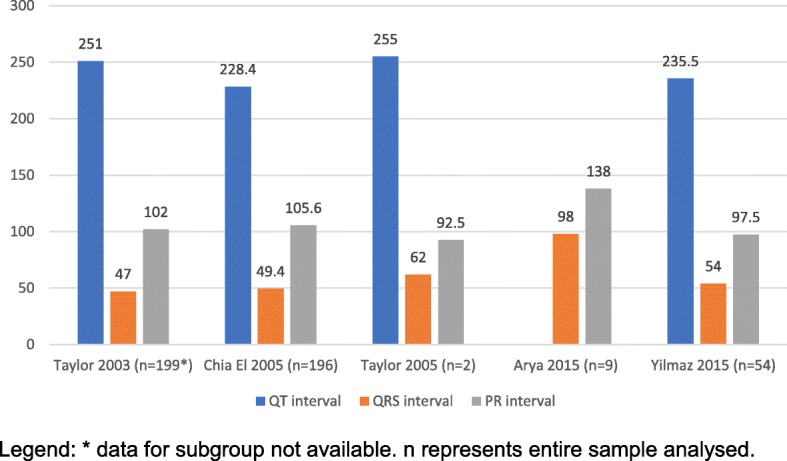
Cardiac time intervals for early pre term (≤32 weeks). Legend: * data for subgroup not available. n represents entire sample analysed

**Fig. 4:**
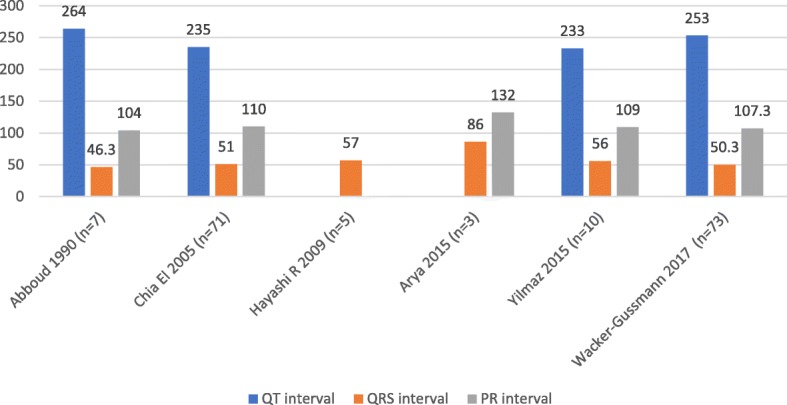
Cardiac time intervals for late pre term (32- 37 weeks)

**Fig. 5:**
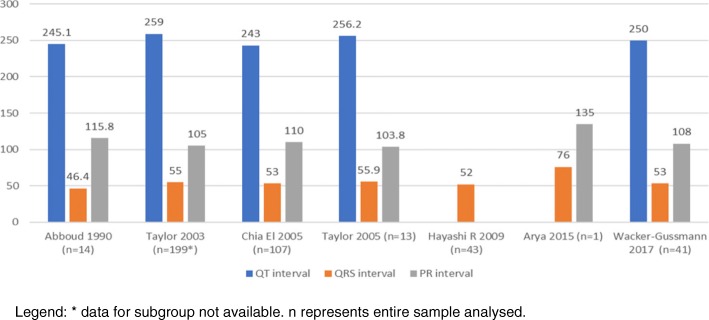
Time intervals for term pregnancies (≥37 weeks). Legend: * data for subgroup not available. n represents entire sample analysed

Table 2 provides the CTI parameters extracted manually from the studies in included in the review. Figures 3, 4, and 5 illustrates this across various gestations as grouped by the WHO classification: early pre-term (≤ 32 weeks, Fig. 3), moderate to late pre-term (32–37 weeks, Fig. 4), and term (37–41 weeks, Fig. 5).

**Table 2:** Cardiac Time intervals as per the WHO classification

Study	N	Early pre– term (≤ 32 weeks)						Late pre– term (32–37 weeks)						Term (≥ 37 weeks)					
(ms)		QT	CI	QRS	CI	PR	CI	QT	CI	QRS	CI	PR	CI	QT	CI	QRS	CI	PR	CI
Abboud et al. (1990) [20]	21							264	224–302	46.3	44–49	103	88–117	245	225–265	46.4	43–50	115.8	91–141
Taylor et al. (2003) [24]	199	251	289–304	47	61–65	102	110–114							259	306–321	55	70–75	105	133–142
Chia et al. (2005) [22]	374	228.4	219–239	49.4	45–54	105.6	99–118	235	230–240	51	48–52	110	107–113	243	239–245	63	51–55	110	108–112
Taylor et al. (2005) [25]	15	255		62		92.5								256		56		103.8	
Hayashi R et al. (2009) [23]	48									57						52			
Arya et al. (2015) [21]	13			96		132.8				85		128				76		135	
Yilmaz et al. (2015)	64	235.5		54	47–57	97.5	91–105	233		56	49–63	109	89–110						
Wacker Gussmann et al. (2017) [26]	117							253	247–259	50.3	49–51	107.3	103–110	250	246–254	53	52–54	108	104–112

CI- 95% Confidence interval

### Signal attenuation

Several studies also note a decrease in signal acquisition between the 27–32 weeks of gestation. This has been explored in detail in Table 3.

**Table 3:** Loss of signal between 27 and 32 weeks of GA

Study	Signal Acquisition Loss (%)
Abboud et al.,1990 [20] (*n* = 21)	None reported
Taylor et al.,2003 [24] (*n* = 199)	12.4%
Chia El et al.,2005 [22] (*n* = 178)	PR = 45.9%, QRS = 19.7%, QT = 27.9%
Taylor et al.,2005 [25] (*n* = 15)	None reported
Hayashi R et al.,2009 [23] (*n* = 48)	40% for *<* 36 weeks
Arya et al.,2015 [21] (*n* = 50)	33%
Yilmaz et al.,2015 (*n* = 64)	15%
Wacker-Gussmann et al.,2017 [26] (*n* = 117)	44%

Individual CTI success rates were evaluated in certain studies as well. Arya et al. noted a significant reduction in signal acquisition between 25 to 30 weeks GA [21]. Chia et al. focused on the group between 27 and 32 weeks and noted CTI acquisition to be significantly lower as follows: P wave (54*.*1%), PR (54.1%), QRS (80.3%) and T wave (70*.*8%) [22]. Chia et al. postulated that P wave acquisition rates remained low due to the low P wave amplitude.

Similar findings were echoed by Wacker-Gussman et al. who illustrated the following acquisition rates at 32 weeks (*n* = 18): P wave (44%), PR (44%) and QRS (44%) [26]. Taylor et al. (2003) demonstrated that 84% (31/37) of separation failures occurred between 27 and 36 weeks [24].

### Temporal relationships

Some studies demonstrated a directly proportional relationship between the P wave duration and GA [22, 26], while others showed this correlation between PR interval and GA [22, 24, 27]. A similar relationship was also observed between the QRS duration and GA as well in select studies [22, 24, 27].

### T wave acquisition

In particular, across most studies, the T wave acquisition rates remained consistently low (22–79%) [22, 24, 27]. Taylor et al. (2003) however, demonstrated an increased detection rate of T wave with advancing gestation OR 7.5 (CI 3*.*5–16*.*3) [24]. These findings were echoed by Yilmaz et al. who found increased T wave detection above 34 weeks in particular [27].

## Discussion

### The fetal electrocardiogram (FECG)

**Fig. 6:**
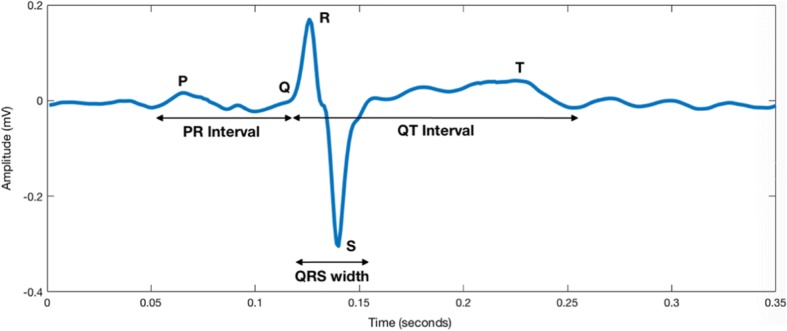
Cardiac time intervals illustrated on a fetal ECG beat. The following beat was extracted from the Physionet database. This was aresult of 10 averaged beats after the maternal ECG was cancelled

The FECG morphology, as illustrated in Fig. 6, is similar to that seen in the adult and contains the P wave, QRS complex and T wave. Fetal cardiac physiology is functionally different from its adult counterpart. In the fetus, the right ventricle plays the dominant role in perfusing the systemic circulation. As a result, the fetal cardiac axis points towards the right in the fetus in contrast to the left-sided deviation in the adults [28]. This difference in orientation results in the FECG appearing morphologically different from an adult ECG [6]. The FECG waveform is processed to provide a familiar ECG visualization to the clinician.

### Cardiac time interval (CTI) analysis

A variety of automated computational methods which have been developed for enhanced analysis of the FECG.

#### Signal detection

The non-invasive nature of the NIFECG relies on signal acquisition from the maternal abdomen. The raw signal consists of the FECG buried within the maternal.

ECG (MECG) signal and environmental noise, such as the uterine muscle activity (UA). Importantly, between the 27th and 32 weeks of pregnancy, the vernix caseosa coats the fetus’ skin and acts as an electrical insulating layer, reducing the efficacy of acquiring the abdominal FECG signal. Signal detection methods have vastly improved over the last 20 years and available technologies at present allow for the detection of the FECG complex from the acquired raw signal [29]. The improvements include and are not limited to new electrode materials (with greater conductivity and skin adhesion), enhanced magnetic shielding of the electronic system, and enhanced electronic designs for noise reduction. The T wave in particular has been known to have lower detection levels due to its weaker signal and distortion by low frequency background noise. As demonstrated by Taylor et al., detection rates of CTIs in the term fetus tend to be more consistent (92%) [25]. Of note are also the presence of physiological conductors and insulators which enhance and attenuate the fetal ECG respectively. Amniotic fluid is an example of a conductor which helps propagate the fetal ECG from the fetal heart to the maternal skin. The vernix caseosa is a sebaceous, protective coating that forms between the 27th to 32nd weeks and persists partially till the 37th week of gestation where it fully dissolves. The vernix caseosa acts as an electrical shield, attenuating the fetal ECG signal. During this period however, a fetal ECG can still be observed non invasively on the mother’s skin as the fetal ECG leaks through the current pathways such as the umbilical cord, oronasal cavity and holes in the vernix caseosa [19, 30].

#### Signal enhancement

Signal enhancement could be broken down into two distinct steps. Firstly, pre-processing allows the signal to be observed in a suitable frequency range, eliminating artifacts and unwanted features. Secondly, enhancing the FECG and attenuating the maternal ECG is key to accurate FHR and CTIs calculations.

One key function of pre-processing is to narrow the frequency range of the acquired signal. The frequency band would be dependent on the features that need to be seen. If the ST segment needs to be analysed or observed, a lower bound of 0.05 Hz would be needed. If the ST segment is not a concern, for example in ambulatory monitoring of ECG, then 0.5 Hz is a commonly used lower cut off. Devices have commonly used an upper frequency bound of 250 Hz to 1000 Hz which is more than sufficient given the spectrum of the ECG features can be observed below 80 Hz. Therefore, by Nyquist sampling, any sampling frequency above 160 Hz should capture the entire ECG signal with all its features. The upper bound ensures that the sharpest features of the signal can be observed whilst attenuating high frequency noise. The lower bound is meant to cut-off as much baseline drift as possible without compromising low frequency components in the NIFECG signal. Line noise, defined specifically at 50 or 60 Hz, can be cancelled out by a notch filter, which eliminates specific frequencies without altering the rest of the signal. The notch filter frequency has no bearing on the upper or lower bound of the frequency band.

The NIFECG signal, being dominated by the maternal ECG, would require enhancement and processing before it could be utilised for FHR or fetal CTIs [5, 15] . This would mean attenuating the maternal ECG to allow the details of the FECG to be observed. The MECG and FECG occur as independent events and therefore attenuating the maternal signal would not comprise information in the FECG if done accurately.

A variety of techniques exist, such as adaptive filtering, Kalman filtering, Bayesian inference techniques, and de-noising methods [31]. Adaptive and Kalman filtering involve multiple observations of the signal, extraction of the FECG waveform, and suppressing the maternal ECG signal through inference or using the maternal ECG lead as a template. In de-noising methods, the NIFECG signal is decomposed into multiple components and the non FECG components, such as Electromyograms (EMG) and MECG, are set to zero. In the case of true synchronicity in timing of the MECG and FECG, a fetal ECG embedded within a MECG would deform the morphology of the MECG signal. Therefore, methods such using a reference maternal lead containing only MECG to train the algorithms to recognise only the FECG, or mathematically transforming the NIFECG into a space where fetal and MECG can be clearly differentiated would be the solution. If FECG happens to be embedded with the maternal QRS complex, that particular beat may be discarded due to the excessive distortion cause by the large amplitude maternal QRS complex. Ideally, FECG should be extracted from isoelectric portions of the MECG, where there are no MECG features to corrupt the FECG.

#### Waveform detection

Once the signal enhancement is complete, the FECG signal can be analysed for CTIs given that the PQRST features will be more prominent. However, the FECG signal is still within the noise band of the acquisition devices and has to be enhanced further before the PQRST features can be reliably detected. To achieve further denoising, several beats are averaged which provides a smoothing effect on the signal at the cost of losing minute details on the FECG. The averaging ranges from 10s up to 2 min or up to 1000 beats. After averaging the beats, the PQRST are detected by either identifying the QRS as a high frequency feature and P and T waves as low frequency features. There will be loss of information given beat to beat variability of cardiac events but averaging cardiac cycles makes the assumption that the ECG signal is quasi stationary over short time windows, meaning the features of each cardiac cycle within that short time window remain consistent. Different studies have used different time windows and there is no standard. This assumption must be made and may fail in the event of ectopic beats or paroxysmal arrhythmias. Another method is to differentiate the waves using their slope, amplitude, and width as per the Pan Tompkins algorithm]. The Pan Tompkins algorithm consists of 2 learning phases and one detection phase. The learning phases determines the thresholds and limit values and the detection phase produces a pulse for each QRS complex. [32].

#### Techniques for fetal ECG enhancement and the effects of noise

The sensitivity of the FECG needs to be considered given the weakness of the signal in comparison to the maternal ECG. By over filtering the signal, several features and CTIs in the FECG may be distorted and become unreliable. When pre-processing, the lower bound of the filter will affect the ST segment. If a filter more than 0.05 Hz is applied, the ST segment’s morphology will be affected and become unreliable for diagnostic purposes. This presents a trade off as filtering in the frequency domain for baseline wander causes distortion of the ST segment. A way around this and subject of potential research would be to identify new transforms where ST segments are preserved whilst eliminating low frequency noise.

When the FECG beats are averaged to remove residual noise, the number of beats used will have an effect. Though the more beats used the cleaner the signal obtained, it also means the P, QRS and T waves will widen and hence provide inaccurate CTI calculations. The CTIs should therefore be viewed in relation to the length of averaging. The MECG and FECG signals are quasi-stationary, which means that beats have similar characteristics over a short period of time whilst the heart reacts to changes in stimuli or physiological conditions. This would mean the widths of the waves as well as relative positions of the P, QRS and T waves with respect to each other would change with varying number of beats used for averaging.

### Clinical correlation of CTIs

The focus of this paper will be in relation to the temporal intervals for the FECG, as illustrated on Fig. 6. For the purpose of the following discussion, it must be borne in mind that evidence discussed below is mitigated by the technological limitations applicable to the era in which they were carried out. Furthermore, all data presented below has been derived utilising the FSE. As such, caution should be applied in loosely comparing these findings to modern signal acquisition techniques as well as the NIFECG. Additionally, the number of subjects should also be taken into account when interpreting the findings of individual studies. For instance, Arya et al.*..* (*n* = 20) demonstrated no correlation between all CTIs and GA.

#### P wave

This parameter refers to the time interval between the onset and end of the P wave. There has been demonstrable evidence to correlate an increase in P wave duration with cardiac size from 17 weeks of gestation [33] . These were similar to findings in Wacker-Gussman et al. (*R* = 0*.*2; *P <* 0*.*05) and Chia et al [22, 26]*.* .

In screening for hypoxia, the utility of P wave duration remains equivocal and unproven. Murray demonstrated P wave duration prior to delivery had a negative correlation with umbilical vein noradrenaline levels (*r* = − 0*.*4, *p <* 0*.*03) [34]. Conversely though, Jenkins et al. produced results showing no correlation between P wave duration and hypoxic and non-hypoxic fetuses as well [35].

From a technical point of view, there are a number of factors which complicate the process of detecting and interpreting the P waves utilising NIFECG. Firstly, its amplitude is low making the signal detection difficult transabdominally. In addition, the width of the P wave would be affected by the number of beats used in the waveform averaging process. The larger the number of beats, the wider the waveform would become and this would make the calculation of the P wave width unreliable as well.

In this context, the available evidence does not seem to demonstrate a role for utilising the P wave in screening for fetal hypoxia. Taking these technological limitations into account however, further research utilising NIFECG would possibly clarify its role in CTI analysis.

#### PR and RR interval

This refers to the duration between the onset of the P wave and onset of the R peak which denotes the conduction times from depolarisation of the SA node to conduction through the AV node and Bundle of His. The PR interval tends to be longer in male fetuses in comparison to female fetuses presumably due to weight differences [36] . A temporal relationship between PR interval and GA was also noted by Chia et al., Taylor et al. and Yilmaz et al. in their study [22, 24, 27].

In animal models, studies have demonstrated the lengthening of the PR interval with hypoxia [37, 38]. In the lamb model specifically, PR interval and RR interval lengthening were demonstrated during aortic occlusion in sheep. This was hypothesised to be secondary to a vagal response – since it could be obliterated with the administration of atropine and was not reproducible in premature lambs which do not demonstrate advanced baroreceptor and chemoreceptor responses [39, 40].

In humans however, the PR interval has demonstrated paradoxical results in comparison to the animal model. Murray demonstrated in labouring women that there was no significant change in the mean PR interval through the course of labour. In 59% however, shortening of the PR interval was demonstrated in the last hour of labour but this was within the standard error of measurement (13%). This subgroup though demonstrated a weak correlation (*r* = 0*.*2) with umbilical cord gas acidemia [39]. Mohajer et al. also showed a 10% shortening of the PR interval from baseline of compromised fetuses which was however, not statistically significant [41]. In a separate study, he also demonstrated a correlation of the PR interval and umbilical artery pH and lactate (*r* = − 0*.*38, *p <* 0*.*01 and *r* = 0*.*36, p *<* 0*.*01) expressed as a ratio index (RI) [42].Physiologically, this could be reflective of the predominant role of catecholamines in the latter stages of labour which influences and delays the conductance of the electrical signal through the AV node.

As such, the role of the PR interval in screening for hypoxia remains unproven and further studies in human would be useful in clarifying its role and the physiological mechanism, if any, in screening for hypoxia.

Several authors have also demonstrated a physiologically inverse correlation between the PR interval and RR interval which becomes positive with evolving acidosis [15, 33, 34, 42, 43].Where the interaction remains continually positive above 20 min, an increased risk of acute fetal compromise has been demonstrated as well [34]. The theoretical basis of this stems from the differential response of the SA node and AV to evolving hypoxia. A vagal cause of this remains unlikely as similar responses can be elicited in mature lambs which have been pre-medicated with atropine [43]. During mild hypoxemia, catecholamine levels become elevated resulting in a concomitant increase in fetal heart rate and a shortening of the PR interval - thereby sustaining the negative relationship between both variables. As the hypoxemia gets progressively worse, the highly oxygen dependent slow sodium channels in the SA node are affected before the fast sodium channels present at the AV node, thereby resulting in a compensatory fall in heart rate and RR interval widening. The catecholamine levels though, continue to rise in line with the evolving hypoxemia thereby continually shortening the PR interval. These synergistic changes would therefore inverse the relationship between both variables to make it positive [39, 44] .

To complicate matters however, Luzietti et al. demonstrated similar inversions in the PR-RR relationship which occurred in all bradycardias below 40 bpm [45]. Westgate et al. further demonstrated similar changes in the relationship during the first 30 min of repetitive umbilical cord compressions in term lamb which however, reverted to negative even in the setting of severe hypoxia. This made them question its discriminative ability and cautioned against potentially misdiagnosing a severely hypoxic fetus as being normal [46].

Based on these findings, two parameters were subsequently trialled in clinical studies in the hope of augmenting existing fetal surveillance parameters. The first was the conduction index (CI) which was a derivative of the Pearson’s correlation between the PR interval and the FHR and calculated every two seconds. Fetal distress was suspected based on a positive relationship establishing for longer than 20 min. The second was termed the ratio index (RI) which was a Z transformed product of the interaction between the FHR and PR interval across the total duration of monitoring undertaken across labour which was computed every 10 s to look for chronic fetal decompensation. Utilising a cut-off of *>* 4% provided a high specificity of 95.5% and accuracy of 89.4% for cord acidemia [42].

Clinically, Reed et al. were the first to assess the utility of PR interval analysis. In their study the addition of PR interval assessment reduced the utilisation of fetal blood sampling (FBS) from 85.5 to 26.8% which resulted in a 4% reduction of missed acidosis at birth [47] . This was followed by a randomized controlled trial (RCT) carried out by Wijngaarden et al. women were randomised to either routine CTG and labour management or CTG monitoring and PR interval analysis. In the latter, if two of the 3 criteria (abnormal CTG, *R >* 4% or CI positive for *>* 20 min) were present, FBS or delivery were to be undertaken at clinician discretion. The study found a significant reduction in the group with PR interval analysis of the number of FBS undertaken, the likelihood of an abnormal FBS, missed cord acidemia at delivery and assisted deliveries for presumed fetal distress [44]. These findings were subsequently followed on by a larger multicentre RCT carried out by Strachan et al. The findings of the study however only demonstrated a non-significant reduction in the group with time interval analysis included [63 (13%) vs 78 (16%)] and no significant difference in identifying cord acidemia or unsuspected cord acidemia [48].

In this context, the available evidence does not seem to demonstrate a significant role for utilising the PR interval in screening for fetal hypoxia. Taking the technological limitations into account however, further research utilising NIFECG would possibly clarify its role in CTI analysis.

From a technical point of view, the widening of the signal due to averaging of the beats will not have an impact on PR measurement since the ratio of PR and RR is considered rather than an absolute measurement. However, if CIs or RIs are being used, the averaging window needs to be carefully considered. For CIs & RIs, since a correlation is calculated every 2 and 10 s respectively, the signal averaging window should not exceed those values.

#### QRS duration

The QRS duration is a measure of the QRS complex and correlates with the time taken for ventricular depolarisation. The QRS duration is longer in males in contrast to females and is directly correlated with ventricular mass and advancing gestation [2, 8, 49]. These findings were mirrored in Chia et al. and Taylor et al. 2003 [22, 24]. There have been suggestions of its utilisation as a surrogate marker for fetal growth and the diagnosis of fetal growth restriction [49, 50]. Pardi et al. suggested that serial measurements would provide a sensitivity of 81% and specificity of 93% in detecting growth restriction if performed serially [37]. Brambati et al. also investigated its utility in women with haemolytic disease of the newborn and noted its ability to discern between fetuses with worsening prognosticating based on a QRS duration greater than four standard deviations above the mean QRS duration for the gestation [51].

From a clinical point of view, the findings regarding the relevance of the QRS complex are mixed. Some authors have demonstrated QRS widening with cord compression [52, 53] . There has however, been no demonstrable link between perinatal outcomes and the QRS duration as these changes could also be demonstrated in normal labours as well [33, 38, 54].

#### QT interval/ QTc interval

The QT interval represent the time taken for depolarisation and repolarisation of the ventricles. The QTc corrects the QT interval for extremes of heart rate. In humans, Oudijk et al. noted in their post hoc analysis of 68 fetuses with acidemia at birth the shortening of both the QT and QTc when metabolic acidosis was present and during variable decelerations between the onset and end of labour. They theorised this to be related to a catecholamine effect [53, 55]. Similar findings were noted in the recipient fetus in TTTS - which exhibits myocardial diastolic dysfunction that suggested its utility in identifying deteriorating ventricular performance as well [55]. Paradoxically however, there has also been evidence to suggest that QT interval instead is prolonged with fetal acidosis [15] .

As such, the role of the QT interval in fetal monitoring is yet to be established or resolved.

### Areas for further research

The following review highlights several areas to address in terms of future research.

#### Large scale prospective studies

The present review has identified the necessity for larger scale prospective trials to establish a reliable set of normal CTIs for fetuses across various gestations. This will pave the way for a reliable reference standard to be established in the field. The values presented here in Table 2 would ideally provide a matrix to build future NIFECG studies upon. Ideally, the studies should be grouped in 4 weekly segments (i.e 24–28 weeks, 28–32 weeks) to increase their utility and accuracy. In addition, there would be virtue in exploring technological consistency and validity across these segments as well. Statistical techniques which would aid in interpreting these between group differences would include and are not limited to the intraclass correlation coefficient (ICC) and regression analysis. In comparing the NIFECG, the FSE would be the reference standards for CTI based information as such data cannot be reliably extracted from the CTG. Research direction should also focus on exploring the performance of the CTIs in screening for fetal hypoxia as well. End points of note for hypoxia can be identified from the discussion section of the following review.

#### Establishing NIFECG databases

Data collected during studies should be combined to form databases to allow investigators in the field to test various algorithms to extract CTIs. Though beyond the scope of this study, Behar et al. provide a reference guide on how to build a standard NIFECG for research purposes which serve as a valuable reference to researchers in the field [31]. This will contribute to conformity and higher quality of data.

#### Technological consistency

As discussed above and as presented in Table 1, the methods utilised to acquire and process the CTIs are varied in nature and can lead to measurement error bias in the CTI values.

In the context of CTI analysis, consistency between signal processing techniques should be established in order to allow for meta-analysis of data. The averaging of the beats and signal filters in particular need to be considered when performing a meta-analysis to ensure the data is treated within bounds that allow it to be judged as similar. The number of beats or width of the window used for signal averaging is important as a large number of beats or large window will lengthen the CTIs and won’t be representative of the quasi stationary nature of the individual beats. Minimal window sizes of less than 5 s would be preferable due to the high variability associated with fetal heart rate.

Signal filters allow for noise attenuation and enhancement of the signal. However these filters can cause phase delays affecting morphology and temporal alignment between the different leads. Also they eliminate various frequencies which again affect the morphology of the signal, which depending on performance, would affect the signal loss and CTI calculation.

#### Improving detection methods

Attention should be directed towards improving or overcoming signal attenuation encountered between 28 and 32 weeks in gestation. This can be overcome by adding leads for the pick up of leaked FECG signals. An increased number of leads, greater signal amplification and robust de-noising techniques would aid in improving signal loss during the 28th to 32nd week period. This approach tackles the problem from a signal acquisition, pre-processing and post processing perspective. The greater number of leads would improve the chances of picking up leaked fetal ECG signals which would be directional based on its source, the electronic amplification enhances the signal at point of acquisition and the robust de-noising would enhance the usefulness of each individual lead.

Another issue lies in the lack of gold standard measurements of CTIs used as benchmarking. This would be necessary to establish how accurate are the CTIs, especially in the case of NI-FECG.

### Limitations

There were several limitations for the following study. Firstly, given the small amount of data published, there was as limited amount of data for analysis. In particular, the study by *Taylor* et al. was utilised in patients in labour. Although all foetuses included in the study were normal and no instances of fetal distress/ hypoxia was mentioned in their study, the effect of labour on the CTIs needs to be taken into consideration as it may have affected our results [25]. Also, the wide variation in CTI acquisition techniques and signal processing did not allow for meta-analysis. This would have been useful for examining temporal relationships between the CTIs. In addition, the studies included in the review were at high risk of bias due to study design as well. Nevertheless, the following studies do still demonstrate the benefit and potential in utilising CTIs in fetal diagnostics.

## Conclusion

In conclusion, NIFECG shows promise as an adjunct diagnostic tool in fetal diagnostics. Larger prospective studies should be directed towards establishing reliable CTIs across various gestations and investigating correlations between the parameters to establish it as an effective screening tool. There is also potential benefit in establishing consistencies in signal processing techniques during a period where much attention is being directed toward this monitoring modality. Furthermore, technologies being developed in the area should aim to address current shortcomings in signal detection to improve reliability and functionality of the methodology.
